# Functional models and extending strategies for ecological networks

**DOI:** 10.1007/s41109-017-0032-5

**Published:** 2017-05-25

**Authors:** Gianni Fenu, Pier Luigi Pau, Danilo Dessì

**Affiliations:** Department of Mathematics and Computer Science, Via Ospedale 72, Cagliari, 09124 Italy

**Keywords:** Ecological networks, Similarity, Network modeling

## Abstract

Complex network analysis is rising as an essential tool to understand properties of ecological landscape networks, and as an aid to land management. The most common methods to build graph models of ecological networks are based on representing functional connectivity with respect to a target species. This has provided good results, but the lack of a model able to capture general properties of the network may be seen as a shortcoming when the activity involves the proposal for modifications in land use. Similarity scores, calculated between nature protection areas, may act as a building block for a graph model intended to carry a higher degree of generality. The present work compares several design choices for similarity-based graphs, in order to determine which is most suitable for use in land management.

## Introduction

In the current society that is the result of centuries of urbanization and industrialization, environmental policies involving the preservation of endangered species and habitats are held as a necessity. The simple approach consisting in the creation of natural reserves with the specific purpose of preserving a target habitat or species has shown its limits, and in the past few decades, there has been a paradigm shift toward the creation of ecological networks, with a focus on the preservation of biodiversity. In the European Union, the Natura 2000 project was established with the goal to create a continent-wide ecological network.

Graph models are a common mathematical tool to analyze and understand network properties in many kinds of networks and complex systems. Modeling choices can be guided by attempts at generalization, or by an interest for a specific point of view. Most infrastructure networks (power grids, roads and railways, etc.) are usually straightforward to represent as a graph, as edges are made to correspond to physical links in existence between the entities represented by nodes; the real challenge in creating a faithful model is that of choosing a measure for edge weights, such that the model can accurately predict the behavior of the underlying network. For power grids, among proposed measures for edge weights are impedance (Cheng et al. 2013) and reactance (Dwivedi et al. 2009). Other complex systems exist, such as social networks and conceptual networks dealing with knowledge representation, for which even the criteria to link node pairs may vary according to analysis goals. For example, in the analysis of a centralized social network, the decision to link people according to whether they are in a contact list, or whether they have recently exchanged messages, may have a strong influence on the resulting graph model and the results of its analysis (Viswanath et al. 2009). Some kinds of infrastructure networks can also be modeled with different approaches; namely, computer networks can be analyzed based on their physical links (Lad et al. 2006), or according to their participation in complex systems of web service orchestration and grid computing (Aymerich et al. 2009).

In the field of ecological landscape networks, the most common approach to building graph models is that of representing functional connectivity, in the sense that the edges in a graph correspond to actual or potential migrations of a species of interest. When possible, data on actual migrations of animals may be derived by tagging and tracking the movement of a sample of the population; alternatively, a method to infer potential migrations is to analyze genetic differences among distant populations (Naujokaitis-Lewis et al. 2013). Graph-theoretic approaches are also possible in studying the relationships among populations, as opposed to the topology of a migration network (Dyer and Nason 2004).

A drawback of the functional approach to building graph models of ecological networks is that assessing general properties of the network requires the analysis of a large number of graphs. This article applies methods from a previous work (Fenu et al. 2016), in which the computation of similarity scores between sites is proposed as a way to build a graph model with a general perspective. In this work, similarity analysis is extended beyond the restricted case of binary vectors, and a refined case study is considered, which takes into account additional constraints in graph generation.

## Ecological networks and graph models

Following the surge of urbanization that has taken place in the past century, the establishment and maintenance of nature protection areas has become an essential part of land management, due to the necessity of a proactive approach in the protection of habitats and species at risk of extinction. Reserves are made up of habitat patches intended as a host for endangered species. Decades of studies and continued endeavor have shown that the effectiveness of reserves is quite limited if patches are not large enough to host a significant amount of population of the protected species, or if reserves are too distant from other suitable habitat patches. Policies for the protection of the environment have thus converged toward the creation of ecological networks, rather than isolated reserves. In an ecological network, each area is intended to contribute to large-scale preservation goals, and efforts are made to ensure a possibility of migration for protected species whenever possible, in order to avoid a deterioration of the gene pool and protect biodiversity (Vimal et al. 2012). Migration can be encouraged with the creation of man-made ‘habitat corridors’ in cases where it is deemed useful; these can be either contiguous or made up of sets of disconnected patches, referred to as ‘stepping stones’.

The ecological network paradigm is being adopted in various parts of the world by the relevant administrative bodies; in the European Union, the project denominated “Natura 2000” is aimed at coordinating the efforts of member states in maintaining a network of nature protection areas with consistent methods and goals throughout the Union. The founding elements of this network are its sites, designated as Special Protection Areas (SPA), as defined in the EU Birds Directive (2009/147/EC), and Special Areas of Conservation (SAC), as defined in the EU Habitats Directive (92/43/EEC). The latter are first proposed as Sites of Community Interest (SCI), and later designated as SACs. A site can hold a designation as a SPA and as a SAC (or SCI) at the same time; alternatively, the boundaries of a SPA can overlap with those of SACs or SCIs. Lastly, sites of the same category can be adjacent to one another.

Local administrations are involved with the management of Natura 2000 sites within their jurisdiction, and can be affected by the presence of sites in their proximity, due to the possible involvement in the creation of habitat corridors. The identification of threats and the proposal of a course of action to eliminate or mitigate them is one of the activities, to which local administrations are to contribute. This requires the consideration of technical, regulatory, and political aspects.

Not unlike other kinds of networks and complex systems, graph models are often used to represent ecological networks mathematically and provide a theoretical basis for the understanding and prediction of network properties. A graph consists of a set of nodes and a set of edges, which may be weighted, i.e. with a numeric attribute associated to them to represent a strength of the link or a cost for traversing it. In this context, a node may represent a site or habitat patch, depending on the desired scale, while edges represent connections. Two different approaches to linking nodes are possible: one is the structural approach, consisting in drawing edges and assigning weights to reflect the influence on migrations carried by existing geographical features, acting as obstacles or connecting elements; this approach has not been successful, due to the difficulties in assessing edge weights in a meaningful manner. Instead, structural connectivity is more commonly analyzed with Geographic Information System tools. The more successful approach to building graph models is that of representing functional connectivity, i.e. actual or potential migration flows, with special reference to a target species (Urban et al. 2009). Data on the migration of animals may be obtained by tagging individuals and tracking their movements; in situations where this is not possible, or to extrapolate data on plant dispersal, a way to infer whether migration may have occurred between different areas is to analyze and compare the genetic pools of samples of populations taken from each area. When raster data is available for the relevant portion of land, and patches can be associated with a resistance value for the target species, circuit theory can also be used to predict migration paths (McRae et al. 2008).

In the analysis of a complex network, statistical properties of graph models are extrapolated and compared, whether with those of other networks or common reference models; among the most common features for analysis are node degree, shortest path length, and indices such as the clustering coefficient, which expresses the degree of redundancy of links, and the betweenness centrality index, used to rank nodes according to their occurrences in shortest paths. The meaning and relevance of each index may differ according to the kind of real-world network being represented (Borgatti 2005); interpretations have been proposed for the most common indices in the field of ecological networks (Estrada and Bodin 2008). As a general principle, global indices, calculated for the network as a whole, can be used as a measure of its ‘health’, while local indices, calculated for single network elements, may assist in identifying vulnerabilities in topological networks (Mishkovski et al. 2011). These are often associated to the different degree of resiliency of the network upon removal of specific nodes (Iyer et al. 2013). The comparison of indices calculated for a given network model and for modified versions of the starting model is often useful to predict the effect of modifications on the corresponding real network.

## Similarity of Natura 2000 sites

Among the activities held as part of Natura 2000 project is the collection of data on habitats and species found within each recognized site. Information is periodically gathered on-site and a public data base is kept up to date with reports filed for each site. These reports must conform to a Standard Data Form, released with Commission Implementing Decision 2011/484/EU. The composition of each Natura 2000 site as a set patches of different habitat types, as well as the presence of a set of species, are part of the collected information; however, the form does not establish an explicit relationship between each species and the habitat patch where it is found. This is sensible for the original purposes of the Natura 2000 project, but for data analysis purposes, it has a shortcoming in the fact that no information is stored concerning which habitat type is ideal for each species; this is assumed to be part of expert knowledge, or found in external documents. Consequently, it is not straightforward to represent constraints for the proposal of modifications involving the relocation of species.

In an attempt to address these problems at least partially, it is possible to represent each site as a vector and compute similarity scores of these vectors. This way, the occurrence of a set minimum score for a pair of sites can be considered as a prerequisite for the proposal to add an edge to the network, to reflect the proposal to establish a habitat corridor. In order to simplify the choice of a threshold, it is preferable to adopt a similarity measure that takes values within a set range; examples thereof are the Jaccard coefficient for binary vectors, and cosine similarity for non-binary vectors.

To compute the Jaccard coefficient for a pair of binary vectors, let *f*_11_ be the number of attributes set to *true* (1) in both vectors, *f*_10_ the number of attributes that are *true* only in the first vector, and *f*_01_ the number of attributes that are *true* only in the second vector; the Jaccard coefficient *J* is given by:

1 $$ J=\frac{f_{11}}{f_{01}+f_{10}+f_{11}}.  $$

This coefficient takes values from 0 to 1, where the extremes represent vectors with no *true* attribute in common and identical vectors, respectively.

Cosine similarity is defined as the cosine of the angle between two vectors with non-zero magnitude from the origin in a multi-dimensional space. This measure is commonly used for the comparison and categorization of text documents, which are represented as vectors by considering an attribute for each keyword, with a value corresponding to their number of occurrences in the document. The following is a simple formula to compute cosine similarity:

2 $$ cos(\mathbf{x}, \mathbf{y})=\frac{\mathbf{x} \cdot \mathbf{y}}{ ||\mathbf{x}|| \, ||\mathbf{y}||},  $$

where **x** and **y** are vectors, **x**·**y** is their scalar product, and ||**x**||, ||**y**|| are their magnitudes. Cosine similarity may take values from −1 to 1 for arbitrary vectors, or from 0 to 1 for pairs vectors with positive attribute values.

If the set of species or the set of habitats are used as the list of attributes, vectors to represent Natura 2000 sites can be built from data collected as part of the project activities. More specifically, the attributes of a vector can be made to correspond to identifying codes for each species of interest, or each reported habitat. For example, to build a binary vector from the habitats in a site, each attribute corresponding to a habitat code is set to 1 if the habitat was reported as found within the site, or 0 otherwise. To build a non-binary vector, the value of each attribute expressing a habitat type can be assigned according to the area extension covered by that habitat type within the boundaries of the site, so long as data is consistently available.

Site vectors can also be built with the integration of an external data source. The CORINE program (Coordination of Information on the Environment) provides a set of standardized land use codes, which can make up a useful set of vector attributes, and for which it is easy to find a compatible data source in the form of patch boundaries. The intersection of these land patches with Natura 2000 sites can be computed to populate vector attributes, both for binary and non-binary vectors, according to the same principles that apply to those based on habitat codes. Land use types (referred to as CORINE Land Cover codes or CLC codes) are categorized in a hierarchical manner, with five levels of increasing detail; a vector based on land use data has attributes corresponding to CLC codes of a chosen reference level (for example, at level 3, different codes can differentiate between broad-leaved forests and coniferous forests, or natural grasslands, etc.).

In this study, the open source QGIS software suite (QGIS Development Team 2009) was used to compute intersection of Natura 2000 sites with land patches from public data made available by the Region of Sardinia. Level 3 codes were used, as the fourth and fifth levels of detail were not available consistently in the dataset.

An advantage in integrating an external source for land use data is that areas outside of Natura 2000 sites may be covered, making it possible to compute a similarity score between a site and an arbitrary area, such as that of a proposed contiguous corridor, in an approach combining graph-theoretic approaches and GIS functions (Pinto and Keitt 2008). This is also useful to limit the effect of missing data, which can be detrimental to the analysis (Naujokaitis-Lewis et al. 2013).

## Case study

To provide a test case and illustrate the method of analysis, the subset of Natura 2000 sites found in Sardinia is considered. At the time of data collection, the administrative region including Sardinia and the minor islands in its surroundings has a total of 124 sites, counting those designated as SPA, those designated as SCI, and those with both designations (no SACs have been designated yet in this region). The number of sites located on the main island of Sardinia is 107; a total of 7 sites, including 3 sites within the main island, have to be excluded due to missing data on land use types.

Ultimately, in order to provide a consistent test case with full availability of data, and disregarding minor islands in order for the considerations to be applicable to land animals, 104 sites can be considered, and graphs are to be created from the same set of 104 nodes, each representing a Natura 2000 site, designated either as a SPA or a SCI, or both. If the boundaries of a SPA and a SCI are intersected, two nodes are created, but the sites are considered to be at zero distance from one another. In every graph instance, pairs of nodes with a geographical distance greater than a set threshold (30 Km) are never linked; distances are calculated between boundaries on a map projection, and are to be treated as an approximation, but the amount of error introduced by projections can be considered acceptable for this study. SQLite with the Spatialite extension was used to compute geographical distances. The open source Cytoscape suite (version 3.4.0) was used for graph visualization (Shannon et al. 2003) and analysis, through the native NetworkAnalyzer plugin (Assenov et al. 2008).

**Fig. 1:**
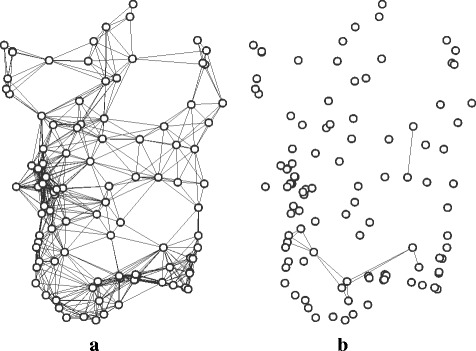
Graph models of Natura 2000 sites in Sardinia. **a** Raw-distance graph. Edges link pairs of nodes with a geographical distance up to 30 Km between boundaries. The position of each node roughly corresponds to the coordinates of the site centroid. **b** Full single-species graph for *Cervus elaphus corsicanus* (species code 1367). Edges correspond to a subset of the edges in the raw-distance graph; each edge links pairs of nodes with the target species reported to be present in both

The graph with 104 nodes, each corresponding to a Natura 2000 site, in which all pairs of nodes within the set geographical distance to one another are linked, shall be referred to as the *raw-distance graph* (Fig. 1 a). The graph thus built with a 30 Km distance threshold has a total of 706 edges.

A way to infer a functional model of the network with reference to a single species is to take the subset of nodes in the raw-distance graph corresponding to sites where the target species has been reported to be found, and link node pairs with an edge if their distance is below the threshold. This can be referred to as a *single-species graph*. Alternately, it may be sensible for comparison purposes to build the graph on the full node set from the raw-distance graph. In this case, node pairs are linked if the sites are within the distance threshold and the target species has been reported in both sites. This can be referred to as a *full single-species graph* (Fig. 1 b). As part of this study, full single-species graphs were built for all species listed in Directive 2009/147/EC and Annex II to Directive 92/43/EEC that were reported to be found in Sardinia (a total of 131 species), keeping the same distance threshold, as a way to establish a frame of reference for the comparison with other proposed graph models. Conversely, if the purpose is to build a functional graph model, the distance threshold ought to be adapted according to the species.

In order to represent the state of the network with a more general point of view, site similarity is used as a criterion to link nodes, as opposed to focusing on data concerning a single species. In this kind of graph, a pair of nodes is linked by an edge if their geographical distance is within the threshold and their similarity is equal or greater than a set minimum score. This process is equivalent to the removal of edges from the raw-distance graph, where the similarity is below the minimum score. A graph model built following such criteria can generally be referred to as a *similarity-based graph*, and specifically according to the choice of similarity measure and dataset from which site vectors are built.

Three different vector representation of sites are possible with available data: one in which attributes correspond to the set of species reported to be in a site, from which a *species-set graph* is built; one in which attributes correspond to the set of habitats according to Natura 2000 categorization (*habitat graph*), and one built on the set of level 3 land use codes according to the CORINE program (*land-use graph*). For all three datasets, it is possible to build binary vectors and compute the Jaccard coefficient for pairs of vectors. Non-binary vectors, for which cosine similarity can be calculated, can also be built for habitat sets and land use codes, either by counting the occurrences or by summing the surface area of each land patch. The latter can be expected to bring more meaningful results, though it can be argued that a similar ratio in the number of occurrences can be a legitimate sign of similarity even in cases where the size of patches is not homogeneous, as they may represent a comparable degree of fragmentation of those patches. Lastly, it is theoretically possible to build non-binary vector for species sets by assigning attribute values according to the amount of population of a species in the site; unfortunately, this kind of data is only sparsely available in the dataset. For this reason, only binary vectors are built from species sets in this study.

To provide a common base of reference, a similarity score of 0.5 shall be used as a threshold to build all of the similarity-based graphs. This threshold was chosen while keeping in mind that, for Jaccard coefficients, similarity scores of 0.6 and above turn out to be strong requirements, which remove over 85% of edges for all three vector types (see Table 1). For consistency, the same threshold will be used with cosine similarity, although it can be observed that this measure is more lenient: a 0.6 threshold removes between about 71 and 78% of the edges in the raw-distance graph (see Table 2). In general, the choice for a threshold value should be high enough for the selected edges to represent reasonably similar sites; conversely, an excessive threshold value can result in an inability to select any edges in practical studies.

**Table 1:** Number of edges in similarity-based graphs of Natura 2000 sites in Sardinia, using Jaccard coefficients

Minimum similarity	Land use-based	Habitat-based	Species-based
0.0 (raw-distance)	706	706	706
0.4	323	248	179
0.5	210	174	122
0.6	93	103	55
0.7	33	53	18
0.8	4	35	16
0.9	0	13	12

**Table 2:** Number of edges in similarity-based graphs of Natura 2000 sites in Sardinia, using cosine similarity

	Occurrence-based		Area-based	
Minimum similarity	Land use	Habitat	Land use	Habitat
0.0 (raw-distance)	706	706	706	706
0.4	361	374	289	230
0.5	277	278	242	185
0.6	201	164	184	155
0.7	126	86	132	123
0.8	79	44	97	97
0.9	35	14	56	55

## Analysis of edge hit rates and complex network indices

Network indices extrapolated with the application of complex network analysis on single-species graph are useful to give insight on the network in its current state, and particularly its aptness for the purpose of conservation of the target species. A common example is the identification of bottlenecks or the detection of insufficient redundancy of links. Proposals for land management can be expressed as modifications of the graph model, with a resulting change of its indices; the best proposals can be identified as those that bring the greatest improvement of indices as an effect.

If a network is initially connected, and the addition or removal of nodes is not considered, proposed modifications can fall into one of three categories (Arrigo and Benzi 2016):

- Addition of edges (‘updating’; proposed edges are referred to as ‘virtual edges’). Given that there is a cost associated with the addition of links in the real-world network corresponding to the graph model, this problem consists in finding a set of new links which results in as great a benefit as possible, while respecting budget constraints.
- Removal of edges (‘downdating’). Assuming that each link has a cost of maintenance, and there is some degree of redundancy of links in the network, this problem corresponds to that of finding a set of edges that can be removed, in order to decrease maintenance costs, while keeping the decrease in the efficiency of the network as low as possible. It is also generally assumed that the removal of links should not create disconnected components.
- Rewiring, i.e. removing and subsequently adding one or more edges. This is related to the goal of improving the efficiency of a network, while avoiding a hike in maintenance costs.

A task for land managers involved with ecological networks is that of enhancing the network effect in a set of habitat patches, or finding a way to contribute to doing so in large-scale settings. A possible intervention that may be considered is that of proposing a site for the relocation of part of the population of a species, among those where it has not been reported, particularly if this has the effect of merging components which are not initially connected in the single-species graph model. While the effect of changes can be evaluated in similar ways, this problem does not correspond to those outlined above, as it involves the addition of nodes.

This introduces the task of identifying suitable candidate sites for this purpose. As previously mentioned, the Natura 2000 data base was not designed for the kinds of data analysis that would be helpful in solving this problem; consequently, it is not straightforward to support the proposal of candidate nodes using the dataset as reference. A good candidate site ought to be within a set geographical distance from an already connected node, and host the preferred habitat for the target species, or if the node is to act as a ‘bridge’, at least a suitable set of habitats for a temporary settlement of the species.

### Aptness of site vectors and similarity measures

When data on the suitability of habitats is missing or incomplete, one of the measures of site similarity may provide a way to formalize this criterion, by suggesting that a good candidate should be a site that does not host the target species, but has similar properties to those of a site that does. Similarity-based graphs can be a useful tool to express and visualize this notion: if nodes in a similarity-based graph are marked according to their presence on a single-species graph built with the same geographical distance threshold, then an unmarked node that is adjacent to a marked node in the similarity-based graph can be considered as a candidate.

In formal terms, let *V* be the full set of nodes that represent Natura 2000 sites in a region of interest, and let *G*_*s*_=(*V*,*E*_*s*_) be a similarity-based graph built on *V* with a suitable geographical distance threshold. Let *G* ^′^=(*V* ^′^,*E* ^′^) be a connected component in the single-species graph built on *V* for the target species of choice (*V* ^′^⊆*V*), with the same geographical distance threshold used for *G*_*s*_. If the following conditions are met:

3 $$ i \in V', \quad j \in V, \quad j \notin V', \quad (i,j) \in E_{s},  $$

then *j*∈*V* can be treated as a good candidate node, and (*i*,*j*) is a candidate edge to link *j* to *G* ^′^.

As already pointed out, similarity-based graphs can differ according to three design choices: the dataset from which site vectors are built (e.g. the set of species or the set of habitats), the similarity measure of choice (Jaccard coefficient or cosine similarity), and the threshold value for the similarity score. A simple way to adjust this value is to match it to a desired percentage of kept edges from the raw-distance graph, as explained in “Case study” section, in an attempt to filter nodes that are not relevant, while ensuring that the process is not hindered by a lack of candidate nodes. Conversely, it is nontrivial to determine which dataset and measure provide the best node candidates.

Intuitively, given two similarity-based graphs *G*_*s*_, *G*_*t*_, built with the same threshold values but different vectors and similarity measures, and a number of single-species graphs *G*_1_...*G*_*n*_, built with the same geographical distance threshold used for *G*_*s*_ and *G*_*t*_, it can be argued that *G*_*s*_ provides better candidates than *G*_*t*_ if edges found in the single-species graphs appear in *G*_*s*_ more frequently than in *G*_*t*_. Thus, in this study, in order to measure the aptness of the each similarity-based graph defined earlier, their sets of edges were compared to those of the 131 single-species graphs built for the species of interest for the Natura 2000 project, using the same 30 Km distance threshold.

Results for a few sample species and average rates are reported in Table 3, for similarity-based graphs based on Jaccard coefficients and a 0.5 threshold. Average hit rates for the same graphs and for graphs based on cosine similarity are reported in the second column of Table 4. The other columns in the latter table express a normalized version of the hit rate: considering that the raw-distance graph has 706 edges (*n*), the number of edges of each similarity-based graph is reported (|*E*|), together with its ‘relative density’ defined as the ratio |*E*|/*n*. If a graph were built with |*E*| edges chosen randomly, a higher number of edges would result in a higher expectation of hit rate. Therefore, hit rates should be normalized to the number of edges. In order for results to be easier to compare, they are equivalently normalized to the relative density:

4 $$ \text{Normalized Hit Rate}=\frac{R}{|E|/n}=\frac{R \cdot n}{|E|}.  $$

**Table 3:** Excerpt of the table of hit rates

		Land use-based		Habitat-based		Species-based	
Species code	Edges	Hits	Rate	Hits	Rate	Hits	Rate
...							
6137	160	80	0.5	47	0.29375	27	0.16875
1367	15	9	0.6	8	0.53333	4	0.26667
1373	8	6	0.75	2	0.25	3	0.375
...							
Average hit rate			0.41607		0.44616		0.47376

For each species, the number of edges in the single-species graph is reported. Then, for each similarity-based graph, it is shown how many of those edges are present in the similarity-based graph (hits), and the corresponding rate. The last row reports the average of all hit rates for each similarity-based graph

**Table 4:** Hit rates in similarity-based graphs

Graph	Avg. hit rate	Edges	Relative density	Norm. hit rate
	*R*	|*E*|	|*E*|/*n*	(*R*·*n*)/|*E*|
Land use (Jaccard)	0.41607	210	0.29745	1.39878
Habitats (Jaccard)	0.44616	174	0.24646	1.81027
Species set (Jaccard)	0.47376	122	0.17280	2.74159
Land use (occurrences)	0.55524	277	0.39235	1.41517
Habitats (occurrences)	0.58591	278	0.39377	1.48795
Land use (areas)	0.40881	242	0.34278	1.19263
Habitats (areas)	0.50503	185	0.26204	1.92730

Since each graph has a different number of edges, a ratio of hit rates to relative density is also provided, where relative density is the ratio of edges in the similarity-based graph to edges in the raw-distance graph (*n*)

The species-set graph emerges as the one with the best hit rate among graphs built using Jaccard coefficients, as well as the one with the highest normalized hit rate. This could be expected, as this graph is built from the same data as those of single-species graphs. A more striking result is that normalized hit rates are consistently higher for habitat graphs than land-use graphs.

**Fig. 2:**
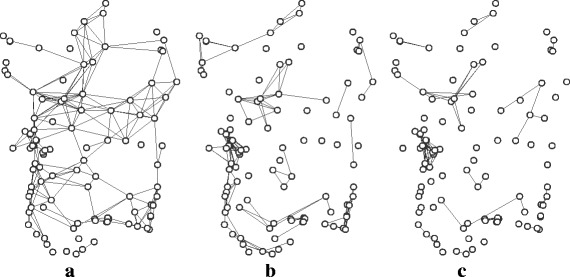
Similarity-based graph models of Sardinian Natura 2000 sites, with a 0.5 similarity score threshold and a 30 Km distance threshold. **a** Based on CORINE land use codes. **b** Based on Natura 2000 habitat codes. **c** Based on species sets

Figure 2 shows the similarity-based graphs built from Jaccard coefficients. It is easily noticeable that the graphs have wide differences, which is confirmed by their different hit rates and network indices. It is interesting to determine whether any pair of similarity-based graphs behave similarly with respect to hit rates. This can be done by comparing the specific hit rates for single species: if the hit rate for two similarity graphs *G*_*s*_, *G*_*t*_ were consistently high for the same set of species, it could be argued that the two graphs express an analogous concept.

In order to perform this evaluation, Spearman correlation indices are calculated between pairs of columns reporting hit rates in Table 3 (and likewise for graphs built on cosine similarity). Results are reported in Table 5 for pairs of graphs based on the same similarity measure; since a species-set graph was built only from Jaccard coefficient, correlations were sought between this graph and the four built from cosine similarities. It is interesting that the species-set graph and the habitat graphs are the only ones for which strong correlations are detected (above 0.77). This is another confirmation that nearby sites with similar habitat sets also host similar sets of species, and is consistent with the fact that land use data originates from a different project. It can be argued that the classification of habitats within the Natura 2000 project is indeed more suitable to describe sites from an ecological point of view, than land use codes are.

**Table 5:** Spearman correlation between sets of hit rates

	Land-use/species	Land-use/habitats	Species/habitats
Jaccard coefficients	0.08351	0.10108	0.80074
Cosine (occurrences)	0.23723	0.40183	0.77293
Cosine (areas)	0.10576	0.20295	0.89486

Species-set graphs are always based on Jaccard coefficients; in each row, a different similarity measure is used for land-use graphs and habitat graphs

### Correlation of network indices

The comparison and search for correlations can be extended to complex network indices calculated for nodes on the various similarity-based graph instances. The question is whether a higher value calculated for each index on a graph corresponds to a higher value for the same index on another. Considered indices are node degree, closeness and betweenness centrality indices, clustering coefficient, and topological coefficient (Stelzl et al. 2005). Correlations are sought between sets of values for the same index on the possible pairs of graph instances built from the same similarity measures or, once again, between the species-set graph built from Jaccard coefficients and the graphs built from cosine similarity.

**Fig. 3:**
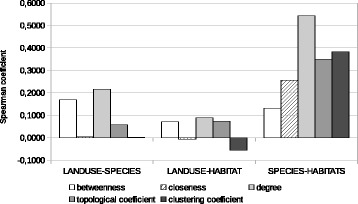
Histogram representation of Spearman correlation of various complex network indices, between pairs of similarity-based graphs built using Jaccard coefficients

Results for graphs based on Jaccard coefficients are reported in Table 6, with a visual representation in Fig. 3. In this case, there is no clear evidence of a strong correlation; however, a moderate degree of correlation can be identified for three indices (degree, topological coefficient and clustering coefficient) between the species-set and the habitats graph. This reinforces the observations that the land-use graph expresses a different concept.

**Table 6:** Spearman correlation of various complex network indices, between pairs of similarity-based graphs built using Jaccard coefficients

Index	Land-use/species	Land-use/habitats	Species/habitats
Betweenness centrality	+0.17010	+0.07141	+0.13077
Closeness centrality	+0.00350	−0.00769	+0.25640
Degree	+0.21587	+0.08945	+0.54399
Topological coefficient	+0.05723	+0.07261	+0.35052
Clustering coefficient	+0.00118	−0.05567	+0.38341

**Fig. 4:**
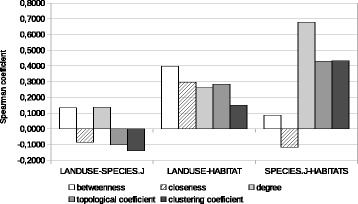
Histogram representation of Spearman correlation of various complex network indices, between pairs of similarity-based graphs. Species-set graphs built using Jaccard coefficients, land-use and habitats graphs built using cosine similarity of occurrences

**Fig. 5:**
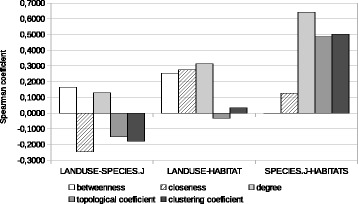
Histogram representation of Spearman correlation of various complex network indices, between pairs of similarity-based graphs. Species-set graphs built using Jaccard coefficients, land-use and habitats graphs built using cosine similarity of areas

The comparison of the species-set graph with land-use graphs and habitat graphs built on cosine similarities, either of occurrence vectors (Table 7 and Fig. 4) or surface area vectors (Table 8 and Fig. 5) leads to similar results: only the habitat graphs show at least a moderate degree of correlation with the species-set graph for some indices.

**Table 7:** Spearman correlation of various complex network indices, between pairs of similarity-based graphs

Index	Land-use/species	Land-use/habitats	Species/habitats
Betweenness centrality	+0.13382	+0.39863	+0.08643
Closeness centrality	−0.08420	+0.29637	−0.11803
Degree	+0.13536	+0.26174	+0.67688
Topological coefficient	−0.10186	+0.28272	+0.43048
Clustering coefficient	−0.13941	+0.15081	+0.43325

Species-set graphs built using Jaccard coefficients, land-use and habitats graphs built using cosine similarity of occurrences

**Table 8:** Spearman correlation of various complex network indices, between pairs of similarity-based graphs

Index	Land-use/species	Land-use/habitats	Species/habitats
Betweenness centrality	+0.16582	+0.25342	+0.00130
Closeness centrality	−0.24685	+0.27600	+0.12489
Degree	+0.12977	+0.31427	+0.64219
Topological coefficient	−0.15156	−0.03243	+0.48585
Clustering coefficient	−0.18044	+0.03439	+0.50126

Species-set graphs built using Jaccard coefficients, land-use and habitats graphs built using cosine similarity of areas

## Conclusions and future work

As complex network analysis becomes an essential tool for land management, the importance of building refined network models that properly represent the state of an ecological network also rises. Current methods of analysis focus on building graph models with a perspective on a single species of interest; their analysis has proven to be useful to determine the aptness of the network in aiding the preservation of the target species, but the evaluation of high-level properties of the network remains a daunting task. Moreover, in the context of the Natura 2000 project, methods for data collection and storage were not originally designed to assist researchers in data analysis, particularly concerning the proposal of network modifications aimed at the improvement of its indices and degree of connectivity.

In this work, graph models based on site similarity are explored and proposed as a way to address this shortcoming. There are multiple ways to build such models, with variations in site attributes from which vectors are built, similarity measure, and score threshold. Several options have been discussed and compared; results confirm that habitat sets from the Natura 2000 dataset and land use data from the CORINE project express different concepts, and the former is more closely correlated with species sets. This represents a challenge for land managers seeking to detect or establish habitat corridors, due to the fact that only land use data is available for areas outside of Natura 2000 sites.

In spite of this limitation, the possibility to apply the methods outlined in this article in the more general case of arbitrary land patches, using vectors based on CLC codes, opens the possibility for future work, as this approach to the evaluation of a potential habitat corridor can be compared with other methods, or be used to complement them.

Further work can also focus on a formalization of an extension of the network updating problem to be applicable in this context. Land use data for patches outside of Natura 2000 sites can be considered as additional information, even while keeping species-set graphs or habitat graphs as reference, in order to take into account the degree of contiguity of potential corridors introduced in the form of virtual edges.
